# Supplementation of *Lactobacillus plantarum* or *Macleaya cordata* Extract Alleviates Oxidative Damage Induced by Weaning in the Lower Gut of Young Goats

**DOI:** 10.3390/ani10040548

**Published:** 2020-03-25

**Authors:** Kai Chen, Yong Liu, Yan Cheng, Qiongxian Yan, Chuanshe Zhou, Zhixiong He, Jianguo Zeng, Jianhua He, Zhiliang Tan

**Affiliations:** 1 CAS Key Laboratory for Agro-Ecological Processes in Subtropical Region, National Engineering Laboratory for Pollution Control and Waste Utilization in Livestock and Poultry Production, Hunan Provincial Key Laboratory of Animal Nutritional Physiology and Metabolic Process, Institute of Subtropical Agriculture, The Chinese Academy of Sciences, Changsha 410125, Hunan, China; chenkia@outlook.com (K.C.); y.liu86@outlook.com (Y.L.); chengyan181@mails.ucas.ac.cn (Y.C.); yanqx14@isa.ac.cn (Q.Y.); zcs@isa.ac.cn (C.Z.); zltan@isa.ac.cn (Z.T.); 2 College of Animal Science and Technology, Hunan Agricultural University, Changsha 410128, Hunan, China; 3 Hunan Co-Innovation Center of Animal Production Safety, CICAPS, Changsha 410128, Hunan, China; ginkgo@world-way.net; 4 University of Chinese Academy of Sciences, Beijing 100049, China

**Keywords:** *Lactobacillus plantarum*, *Macleaya cordata*, inflammation, oxidative stress, goats

## Abstract

**Simple Summary:**

Weaning stress is a serious problem in the goat production industry. This article demonstrates that weaning can induce intestinal oxidative damage, and adding *Lactobacillus plantarum* or *Macleaya cordata* can alleviate the oxidative damage of the lower gut in weaned young goats in an intestinal region-specific way.

**Abstract:**

Weaning usually leads to stress in livestock, which has a negative impact on their growth and development. Research on oxidative stress and inflammation induced by weaning has not been reported in goats. Here, we focused on oxidative stress profile and inflammation status of the lower gut (jejunum, ileum, and colon) of goats. First, we illustrated the status of antioxidant activity and inflammation in the intestine of young goats on pre-(2 weeks postnatal, 2 wk^pn^) or post-(11 wk^pn^, weaning at day 45 postnatal)-weaned period of young goats. Malondialdehyde (MDA) was higher (*p* < 0.0001) in jejunum and ileum of the young goats in 11 wk^pn^ than that in 2 wk^pn^, whereas superoxide dismutase (SOD) activity was lower (*p* = 0.012) in the lower gut of the young goats with 11 wk^pn^ than that in 2 wk^pn^. Furthermore, we intended to explore the protective influence of a probiotic additive (*Lactobacillus plantarum* (LAC) P-8, 10 g/d) and a prebiotic additive (Sangrovit^®^, *Macleaya cordata* (MAC) extract 3.75% w/w premix, 0.3 g/d) on intestinal oxidative stress and inflammation status of early-weaned young goats (average weights of 5.63 ± 0.30 kg, weaned on d 45 postnatal). We observed that LAC reduced MDA in jejunum and ileum (*p* < 0.0001), increased SOD activity in ileum (*p* < 0.01), and increased glutathione peroxidase (GSH-Px) activity in jejunum (*p* < 0.05). Similarly, MAC reduced MDA contents (*p* < 0.0001), increased SOD activities (*p* < 0.01) in both of ileum and jejunum, and increased GSH-Px activity (*p* < 0.05) in jejunum. However, there were no differences in feed intake, average daily gain, inflammation parameters (interleukin 2 and interleukin 6), and colon oxidative stress profile (MDA, SOD, or GSH-Px) among treatments. These results provide evidence that weaning induces oxidative damage in the lower gut of young goats, and the oxidative damage in the small intestine can be reduced by adding the addition of LAC or MAC in diets depending on the region of the lower gut.

## 1. Introduction

Weaning stress is still a long-standing challenge for the livestock productivity and health. The critical period in early life, around pre- and postweaning, results in digestive problem and diarrhea due to colonization of pathogenic bacteria in the gut [1]. Moreover, weaning switches the diet of young mammals from a liquid feed to a completely solid feed, and is commonly associated with the upregulation of proinflammatory cytokine and overproduction of reactive oxygen species (ROS) [2]. Increased oxidative stress and inflammation induced by weaning in mammals have been reported [3,4,5]. Strategies to mitigate the adverse effects of ROS and inflammation include nutrient regulation and feeding management.

Genus *Lactobacillus* (LAC) used as a probiotic additive to avoid the imbalance of redox homeostasis has been widely investigated in other animal species. For instance, Wu et al. [6] have reported that LAC delbrueckii extracellular secretions have the most robust antioxidant activity, which can remove superoxide free radicals. Glutathione peroxidase (GSH-Px) and superoxide dismutase (SOD) can decrease oxidative production by preventing lipid peroxidation [7]. The upregulation of GSH and SOD, and reduction of oxidative stress as a result of lactobacillus supplementation in weaning pigs has been reported [8]. Paszti-Gere et al. [9] have reported that LAC can inhibit intestinal interleukin 8 (IL-8), which confirms the anti-inflammatory properties of the active metabolites by LAC under acute oxidative stress. Furthermore, lactic acid bacteria reduce inflammation caused by bacteria and LPS, by reducing the levels of pro-inflammatory cytokines interleukin 1 (IL-1), interleukin 6 (IL-6), tumor necrosis factor-α (TNF-α), interleukin 17 (IL-17), and interleukin 22 (IL-22), as well as increasing the levels of anti-inflammatory cytokines interleukin 2, 4, and 10 (IL-2, -4, and -10) [10,11,12,13,14].

The probiotic additive of *Macleaya cordata* (MAC) is a plant extract, containing *sanguinarine* and *chelerythrine*. The two components are from benzylisoquinoline alkaloids and beneficial for animal intestinal health [15]. Li et al. [16] have found that MAC can upregulate antioxidant activity. The authors also reported that the extracts of MAC enhance the intestinal barrier function of piglets and have antibacterial and anti-inflammatory effects on the intestinal tract. We hypothesized that weaning induces oxidative damage and inflammation in young goats, and the inclusion of LAC or MAC in the diets of goats improves growth performance and alleviates oxidative damage and inflammation induced by weaning.

The importance of the lower gut in ruminants, especially in young ruminants, has received more attention in recent years. Therefore, the objectives of this study were: (1) to investigate the weaning effects on the oxidative stress and inflammation in the lower gut of young goats and (2) to explore the effects of supplementing probiotics or prebiotics additives on growth performance as well as oxidative stress and inflammation in the lower gut of young weaned goats.

## 2. Materials and Methods

### 2.1. Experimental Design and Animal Managements

For the sake of exploring the protective influence of pre-/probiotics on early-weaned young goats, we conducted two different experiments. The objective of experiment 1 (Exp. 1) was to investigate the weaning effects on the oxidative stress and inflammation in the lower gut of young goats, while the objective of experiment 2 (Exp. 2) was to evaluate the protective function of pre-/probiotics on the hindgut of early-weaned goats. The Animal Care Committee approved all the protocols for the animal procedure at the Institute of Subtropical Agriculture, Chinese Academy of Sciences (ISA-CAS), following the Animal Care and the Use Guidelines of ISA-CAS, China (KYNEAAM-2017-ZLT-0009).

**Exp. 1:** Early-weaned procedure was conducted on postnatal day 45; 17 young female goats (10 preweaned young goats and seven postweaned young goats (weaned at 45 days old)) were used to investigate the effects of weaning on intestinal oxidative stress and inflammation in the young goats. The young goats (Liuyang black goat, local breed) were all from pluriparous ewes and were provided by a local goat farm (Liuyang, Hunan province, China).

After birth, kids were ear-tagged, weighed, and reared together with their mothers until weaning at day 45. Before weaning, all goat mothers were grazed and received a 300 g/d concentrate diet. The concentrate contained (dry matter, DM, basis) of 740 g corn meal, 203 g soybean meal, 13 g calcium bicarbonate, 16 g calcium carbonate, 8 g sodium chloride, and 2 g mineral–vitamin premix per kg. It supplied 11.0 MJ/kg of metabolizable energy and 154 g/kg of crude protein. From birth to day 21, kids received no supplemental feed other than their mother’s milk. From days 22 to 45, the kids were provided goat milk and a starter feed (321 g extruded soybean, 115 g whey powder, 167 g maize flour, 182 g fat powder, 133 g soybean meal, 25 g calcium hydrophosphate, 11 g calcium carbonate, 13 g sodium chloride, and 33 g premix per kg; dry matter basis) for ad libitum intake. After weaning at day 45, kids received a starter diet and fresh grass in a ratio of 6:4 for ad libitum intake without milk. Ten preweaned kids (an average body weight of 2.28 ± 0.0230 kg; means ± SE) with ages between 7 and 14 d and seven postweaned young goats (an average body weight of 5.70 ± 0.957 kg) with an age of 77 days were slaughtered. 

**Exp. 2:** Twenty-one weaned young female goats (weaned at day 45 with an averaged body weight of 5.63 ± 0.30 kg) were provided by the same local goat farm as for Exp. 1. The diets and management of the goats preweaning were also the same as described in Exp. 1. Goats were housed individually, and randomly assigned into one of the three diets: (1) a control diet without a probiotics additive or a prebiotics additive (CON), (2) a control diet supplementing with 10 g/day *Lactobacillus plantarum* P-8 (4.0 × 10^9^ CFU/g, LAC), the detail information of *Lactobacillus plantarum* P-8 strains was described by Bao et al. [17], and (3) a control diet supplementing with 0.3 g/day plant extract (Sangrovit^®^, MAC extract 3.75% w/w premix). The supplementing rates of additive used in this study were suggested by previous studies [15,17]. The goats were fed ad libitum in two equal amounts at 8:00 a.m. and 6:00 p.m., and had free access to water. The amount of a probiotics additive or a prebiotics additive was top-dressed every day to make sure that the goats could consume the entire additive. The feeding trial lasted for 31 days, and daily feed intake and initial and final body weights were recorded to monitor the growth performance of the young goats.

### 2.2. Collection Procedures and Sampling

After slaughtering the goats, the tissues of jejunum, ileum, and colon were separated and washed with 0.9% sodium chloride solution. The samples were then immediately frozen in liquid N_2_ and stored at −80 °C for the measurements of antioxidant activity and inflammation.

### 2.3. Measurements of Antioxidant Activities and Inflammation

The tissues were homogenized in 9 mg/mL precooling sodium chloride and centrifuged at 3000× *g* for 20 min at 4 °C, after which, the supernatant fraction was collected and stored at −80 °C for malondialdehyde (MDA) content and SOD and GSH-Px activities analyses. The MDA content and activities of SOD and GSH-Px were measured spectrophotometrically with a microplate reader (Tecan, Austria) as described by Girotti et al. [18], Flohé et al. [19], and Lawrence [20], respectively. The chemical kits were purchased from Nanjing Jiancheng Bioengineering Institute (Nanjing, Jiangsu, China).

The interleukin 2 and interleukin 6 concentrations were determined using ELISA kits (Beyotime, Shanghai, China) following the instructions of the manufacturer. Briefly, 96 well plates coated with antigoat IL-2 or IL-6 monoclonal antibodies and HRP-tagged antigoat IL-2 or IL-6 antibodies were incubated for 60 min at 37 °C after the samples were added. Wells were then washed five times using the washing buffers, and further, a chromogen solution was added. Plates were incubated for 15 min at 37 °C in the dark, and the reaction was stopped by adding the stop solution. The optical density was measured at 450 nm by a microplate reader. The intra- and interassay coefficient of variations were all below 10%.

### 2.4. Statistical Analysis

Data were analyzed using the MIXED procedure of SAS (SAS Inst. Inc., Cary, NC, USA). The model included treatment (day of age in Exp. 1 and additive in Exp. 2), gut region, and their interaction as fixed effects. If the interaction term was not significant, it was subsequently excluded from the final model. Differences between group means were tested using the Bonferroni adjustment, and statistical significance was declared at *p ≤* 0.05.

## 3. Results

### 3.1. Weaning Effects on Activities of Antioxidant Enzyme and Inflammatory Factor

**Figure 1:**
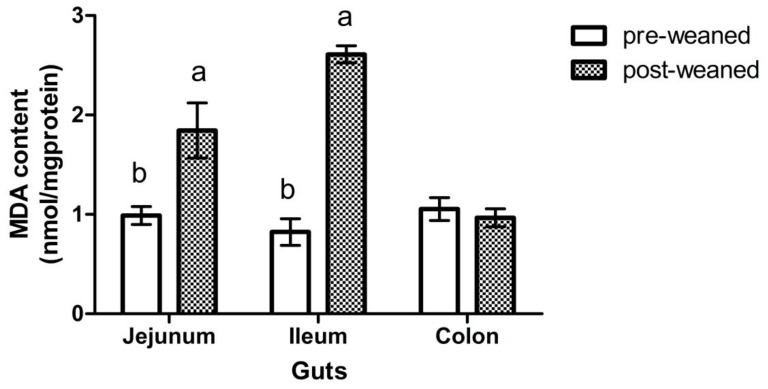
Weaning effects on the content (nmol/mg·protein) of malondialdehyde (MDA) in the colon, ileum, and jejunum of young goats (Exp. 1). ^a, b^ Row means that do not have a common superscript differ (*p* < 0.05).

The MDA contents in the jejunum and ileum for the postweaned young goats were higher than the preweaned young goats (0.99 ± 0.089 vs. 1.84 ± 0.28 and 0.82 ± 0.13 vs. 2.61 ± 0.09 nmol/mg·protein for jejunum and ileum, respectively; Figure 1). There were no interactions (*p* = 0.35) between age and gut region for the activities of SOD and GSH-Px. The activity of SOD in postweaned young goats was lower (*p* = 0.012) than the preweaned young goats (98.8 vs. 56.3 U/mg·protein), and no difference (*p* = 0.829) was observed for the SOD activity among gut regions (Table 1). There was no difference (*p* = 0.846) in GSH-Px activity observed between pre- and postweaned young goats; however, the GSH-Px activity was higher in the jejunum (*p* = 0.037) than the ileum and colon. Moreover, neither age nor gut region effect (*p* > 0.092) was observed on IL-2 and IL-6 concentration in young goats (Table 1).

### 3.2. Effects of Supplementing Probiotics and Prebiotics on Antioxidant Enzyme, Inflammatory Factor, and Growth Performance

**Figure 2:**
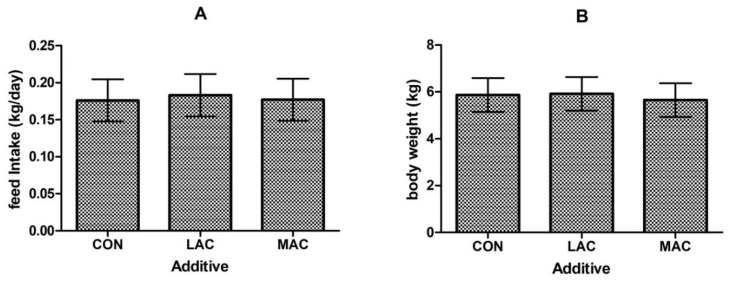
Effects of supplementing probiotics and prebiotics additives on feed intake (**A**) and body weight (**B**) of young goats (Exp. 2). CON = control group; LAC = a control diet supplementing with 10 g/day *Lactobacillus plantarum* P-8; MAC = a control diet supplementing with 0.3 g/day plant extract (Sangrovit^®^, MAC extract 3.75% w/w premix).

**Figure 3:**
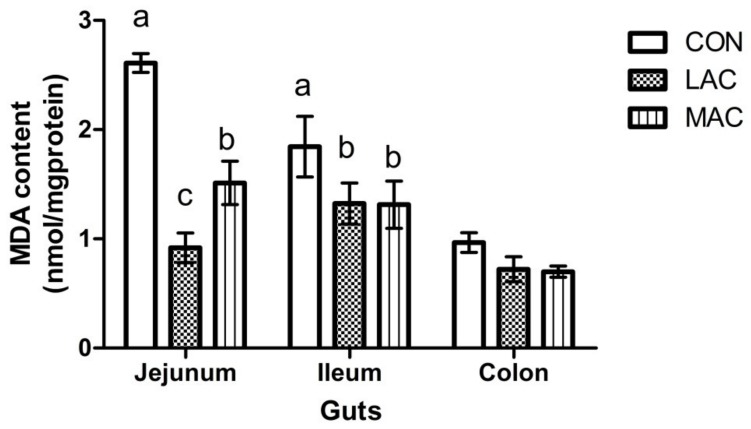
Effects of supplementing probiotics and prebiotics additives on the content (nmol/mg·protein) of MDA in the colon, ileum, and jejunum of young goats (Exp. 2). CON = control group; LAC = a control diet supplementing with 10 g/day *Lactobacillus plantarum* P-8; MAC = a control diet supplementing with 0.3 g/day plant extract (Sangrovit^®^, MAC extract 3.75% w/w premix). ^a, b^ Row means that do not have a common superscript differ (*p* < 0.05).

**Figure 4:**
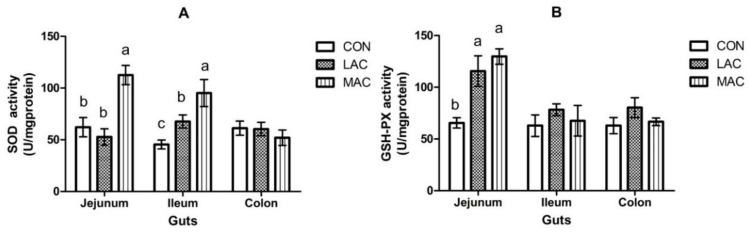
Effects of supplementing probiotics and prebiotics additives on the activities (U/mg·protein) of SOD (Panel **A**) and GSH-Px (Panel **B**) in the colon, ileum, and jejunum of young goats (Exp. 2). CON = control group; LAC = a control diet supplementing with 10 g/day *Lactobacillus plantarum* P-8; MAC = a control diet supplementing with 0.3 g/day plant extract (Sangrovit^®^, MAC extract 3.75% w/w premix). ^a, b^ Row means that do not have a common superscript differ (*p* < 0.05).

No difference (*p* > 0.05) in feed intake (Figure 2A) or final body weight (Figure 2B) was observed among groups. The activity of MDA in the ileum and jejunum of LAC goats was less (*p* < 0.001) than CON goats (Figure 3; 1.84 ± 0.28 vs. 1.32 ± 0.19 and 2.61 ± 0.09 vs. 0.92 ± 0.13 nmol/mg·protein). The SOD activity in the ileum (Figure 4A; 45.41 ± 4.29 vs. 67.68 ± 6.32 U/mg·protein) and GSH-Px activity (Figure 4B; 65.52 ± 5.00 vs. 115.69 ± 14.71 U/mg·protein) of LAC goats were higher (*p* < 0.03) than the corresponding activities in CON goats. Similarly, MDA contents in the ileum and jejunum from MAC goats were less (*p* < 0.001) than that from CON goats (Figure 3; 1.84 ± 0.28 vs. 1.31 ± 0.22 and 2.61 ± 0.09 vs. 1.51 ± 0.20 nmol/mg·protein). The SOD activity in ileum and jejunum (Figure 4A; 45.41 ± 4.29 vs. 95.15 ± 13.05 and 62.20 ± 9.37 vs. 112.60 ± 9.32 U/mg·protein) and GSH-Px activity (Figure 4B; 65.52 ± 5.00 vs. 129.76 ± 7.48 U/mg·protein) in the jejunum from MAC goats were higher (*p* < 0.03) than those in CON goats. However, there were no differences (*p* > 0.05) observed among treatments in MDA, SOD, and GSH-Px in the colon. For the inflammation variables, there were region effects (*p* < 0.05) both on IL-2 and IL-6, with higher activity in the jejunum (Table 2). Supplementation of LAC or MAC additive failed to alter (*p* > 0.19) the concentrations of IL-2 and IL-6 in the young goats (Table 2).

## 4. Discussion

It has been widely reported that weaning in mammals leads to intestinal dysfunction, intestinal microbial disorder, tight connection injury, inflammation, and oxidative stress [21]. Mammalian cells play a vital role in antioxidant defense systems when subjected to extreme stress. Previously, Wei et al. [4] have reported that the activity of ROS is significantly increased with the decrease of the activity of SOD in weaned groups as compared to the preweaning group in the jejunum of piglets. Superoxide dismutase is an antioxidase enzyme that changes the superoxide anion into hydrogen peroxides, and GSH-Px can inactivate peroxides [22]. The imbalance of the intestinal redox status usually restrains the capacity of eliminating ROS, resulting in oxidative damage to the intestinal epithelial cells [23,24]. In addition, MDA is one of the lipid peroxide metabolites and an essential symbol of oxidative damage, which can be enhanced with weaning stress [25,26]. In this study, the higher MDA content with less antioxidant enzymes activity in weaned young goats was expected, which suggests that early weaning can induce oxidative damage in the young goats. Moreover, the previous study in piglets has shown that a significant increase in messenger RNA (mRNA) levels of TNF-α, IL-1β, and IL-6 in the jejunal mucosa was induced by weaning [4]. However, the absence of a significant impact of weaning on IL-2 or IL-6 in the present study may indicate the distinct responses between ruminants and monogastric animals. It has been reported that the change of gastrointestinal inflammatory cytokines is related to the diversity of gastrointestinal microorganisms [27]. Ruminating goats have a unique digestive organ called the rumen, where the microorganisms can convert feed components into readily available sources of energy and protein. Our results suggest that the ruminating goats may be more tolerant of pathogens than monogastric animals.

*Lactobacillus* is an active microorganism that is used as a probiotic additive in livestock [28,29]. The less MDA content and increased SOD and GSH-Px activities in the jejunum and ileum of young goats due to LAC were expected, which suggest a protective effect of LAC in improving animal health. In agreement with our results, it has been reported that LAC increased activities of GSH and reduced oxidative stress in growing lambs. [29]. In addition, Maekawa et al. (2016) have reported a protective effect of LAC pentosus strain S-PT84 against Candida infection of the gastrointestinal tract in mice [11]. The mechanism that LAC can regulate the oxidative stress of animals may be related to modulation of nuclear factor erythroid-2-related factor 2 (Nrf2) signals and suppression of nuclear factor kappa-B (NF-kB) inflammatory pathway [30]. Moreover, it has been reported that *Lactobacillus* plays a significant role in regulating gastrointestinal mucosal immune cells and maintaining a healthy gastrointestinal tract [31]. *Lactobacillus* can produce bactericidal substances and compete for the attachment plaque with pathogens and toxins [31].

It has been reported that MAC can reduce the reproduction of pathogenic bacteria which lead to severe intestinal stress, inflammation, and even death after weaning in piglets [32]. Li et al. [16] have observed an antioxidant ability of MAC using 1,1-diphenyl-2-picrylhydrazyl (DPPH) radical scavenging and β-carotene–linoleic acid assays. The main active components in MAC are sanguinarine and chelerythrine [16], which can induce antioxidant effects [33]. Thus, the improved activities of SOD and GSH-Px in the jejunum and reduced MDA production in jejunum and ileum were expected in our study.

Although decreased MDA with an increased SOD and GSH-Px in jejunum and ileum in young goats for the LAC and MAC groups were observed in this study, the inclusion of LAC and MAC in the diets failed to change the oxidative status in the colons of the young goats. Plausibly, the majority of additives played a role in the small intestine, and colonization of the additives in the large intestine was inefficient [34]. Moreover, since the variety and quantity of microorganisms in the colon are more abundant and numerous compared with that in the small intestine, the added additives are possibly hard to be attached to the intestinal walls, and can be only kept for a very short time [34,35].

Interestingly, we did not observe a difference between the control and LAC-supplemented groups in feed intake and body weight gain. This result was consistent with Whitley et al. [36] that they have found the average daily gain, feed intake, and feed conversion are not influenced by a commercial probiotic supplementation containing LAC acidophilus and *Enterococcus faecium* in meat goats. Conversely, in the study of Sharma et al. [37] using Murrah buffalo calves showed that supplementation of LAC acidophilus improved the growth performance of the calves, which suggests that the effect of probiotic and prebiotic may relate with animal species.

## 5. Conclusions

In this study, weaning increased MDA content and decreased SOD activity in the lower gut of young goats. Supplementation of LAC or MAC reduced the MDA concentration and increased the SOD and GSH-Px activity in the jejunum or ileum of the young goats. However, there were no differences in MDA, SOD, or GSH-Px in the colon of the young goats supplementing with or without a probiotic and prebiotic additive. There was no alternation in inflammation among the goats in this study. The changes in oxidation status indicate that weaning induces oxidative damage in the lower gut in the young goats, and the oxidative damage in the small intestine can be alleviated by adding LAC or MAC to the diets. 

## Figures and Tables

**Table 1:** Weaning effects on the activities (U/mg·protein) of SOD and GSH-Px, and the concentration (pg/mg·protein) of IL-2 and IL-6 in the colon, ileum, and jejunum of young goats.

Item ^1^	Age		SEM	Gut			SEM	*p* Value		
	Preweaned	Postweaned		Jejunum	Ileum	Colon		Gut	Age	Interaction
SOD (U/mg·protein)	98.8 ^a^	56.3 ^b^	8.0	85.1	73.1	74.35	11.9	0.829	0.012	0.701
GSH-Px (U/mg·protein)	62.0	64.4	10.7	88.8 ^a^	53.0 ^b^	47.87 ^b^	12.5	0.037	0.846	0.349
IL-2 (pg/mg·protein)	319.5	327.3	23.9	329.8	267.3	373.2	34.2	0.147	0.855	0.427
IL-6 (pg/mg·protein)	28.9	33.2	2.2	28.3	26.6	38.2	3.2	0.092	0.307	0.683

^a, b^ Row means that do not have a common superscript differ (*p* < 0.05). ^1^ SOD = superoxide dismutase; GSH-Px = glutathione peroxidase; IL-2 = interleukin 2; IL-6 = interleukin 6.

**Table 2:** Effects of probiotics and prebiotics additives on concentrations (pg/mg·protein) of IL2 and IL6 in the colon, ileum and jejunum of young goats.

Item ^1^	Additive ^2^			SEM	Gut			SEM	*p* Value		
	CON	LAC	MAC		Jejunum	Ileum	Colon		Gut	Additive	Interaction
IL2	327.3	294.9	278.0	24.0	381.46 ^a^	254.5 ^b^	266.0 ^b^	26.8	0.020	0.536	0.304
IL6	33.2	26.9	27.1	2.2	38.9 ^a^	23.4 ^b^	25.0 ^b^	2.3	<0.001	0.185	0.397

^a, b^ Row means that do not have a common superscript differ (*p* < 0.05). ^1^ IL2 = interleukin 2; IL6 = interleukin 6; ^2^ CON = control group; LAC = a control diet supplementing with 10 g/day *Lactobacillus plantarum* P-8; MAC = a control diet supplementing with 0.3 g/day plant extract (Sangrovit^®^, MAC extract 3.75% w/w premix).
